# Evaluation of oral microbiology lab curriculum reform

**DOI:** 10.1186/s12909-015-0497-9

**Published:** 2015-12-07

**Authors:** Min Nie, Zhen Y. Gao, Xin Y. Wu, Chen X. Jiang, Jia H. Du

**Affiliations:** The State Key Laboratory Breeding Base of Basic Science of Stomatology, Hubei Province & Key Laboratory of Oral Biomedicine (Wuhan University), Ministry of Education, School and Hospital of Stomatology, Wuhan University, Luoyu Road 237, Wuhan, 430079 Hubei China

## Abstract

**Background:**

According to the updated concept of oral microbiology, the School of Stomatology, Wuhan University, has carried out oral microbiology teaching reforms during the last 5 years. There was no lab curriculum before 2009 except for a theory course of oral microbiology. The school has implemented an innovative curriculum with oral medicine characteristics to strengthen understanding of knowledge, cultivate students’ scientific interest and develop their potential, to cultivate the comprehensive ability of students. This study was designed to evaluate the oral microbiology lab curriculum by analyzing student performance and perceptions regarding the curriculum from 2009 to 2013.

**Methods:**

The lab curriculum adopted modalities for cooperative learning. Students collected dental plaque from each other and isolated the cariogenic bacteria with selective medium plates. Then they purified the enrichment culture medium and identified the cariogenic strains by Gram stain and biochemical tests. Both quantitative and qualitative data for 5 years were analysed in this study. Part One of the current study assessed student performance in the lab from 2009 to 2013. Part Two used qualitative means to assess students’ perceptions by an open questionnaire.

**Results:**

The 271 study students’ grades on oral microbiology improved during the lab curriculum: “A” grades rose from 60.5 to 81.2 %, and “C” grades fell from 28.4 to 6.3 %. All students considered the lab curriculum to be interesting and helpful. Quantitative and qualitative data converge to suggest that the lab curriculum has strengthened students’ grasp of important microbiology-related theory, cultivated their scientific interest, and developed their potential and comprehensive abilities.

**Conclusion:**

Our student performance and perception data support the continued use of the innovative teaching system. As an extension and complement of the theory course, the oral microbiology lab curriculum appears to improve the quality of oral medicine education and help to cultivate high-quality innovative medical talents.

## Background

Oral medicine is a practical and operational subject with its inherent principles and specific characteristics. Oral microbiology is a subject that combines microbiology and oral medicine. Oral microbiology involves oral clinical, microbiology, dental caries, endodontics, and periodontics. Oral microbiology contains oral ecosystem and its influencing factors, dental plaque and biofilm, and methods of oral examination [1–3].

Traditional oral microbiology teaching consists of only lectures without any lab curriculum. For example, students could not understand completely what dental plaque is or what pathogenic bacteria look like. It is ineffectiveto continue the old teaching approach, which is unable to satisfy and adapt to the demands of modern education [4, 5]. Based on the development of oral microbiology and its characteristics, lab teaching should be an important part of higher education to strengthen understanding of theoretical concepts and to cultivate students’ research interest, practice, innovation, and comprehensive abilities [6, 7]. An extension and complement of the theory teaching, it is an indispensable part of education reform at Wuhan University.

The new lab curriculum focuses on plaque collection, cariogenic bacteria isolation, culture, and identification. It fuses basic lab skills with the theory of microbiology, oral histopathology, oral internal medicine, and so on. It also helps students become familiar with endodontic and periodontal disease and is of great significance to perfect education reform [8–10]. We hope the oral microbiology lab curriculum will improve the quality of oral medicine education and cultivate high-quality innovative medical talents.

### Hypothesis

The oral microbiology lab curriculum would contribute to: (1) strengthening students’ understanding of oral biology theory; (2) improving experimental ability; (3) cultivate the students’ potential and interest in scientific research; (4) strengthening oral health education. Generally, students would support the lab curriculum.

## Methods

### Participants and data collection

In Part One of the study, we retrospectively analysed student performance data of the oral microbiology lab sessions in 2009 (*n* = 40), 2010 (*n* = 55), 2011 (*n* = 56), 2012 (*n* = 67), and 2013(*n* = 53). The 5-year database totaled 271 students, including 75 men and 196 women. The oral microbiology lab curriculum was offered to senior students who enrolled in the school in 2006, 2007, 2008, 2009, and 2010. In Part Two of the study, we summarised the 271 students’ perceptions of the oral microbiology lab curriculum in the experiment report.

This entire study was submitted to and reviewed by the Medical Ethics Committee of Wuhan University, which subsequently waived the need for ethical approval. All 271 participants were informed of the aims of the quantitative and qualitative evaluation and consented to participate in the study.

### The oral microbiology lab curriculum

The School of Stomatology at Wuhan University has implemented an innovative oral microbiology lab curriculum for the stomatology professional senior class since 2009. The curriculum includes sampling and processing of dental plaque, isolating and cultivating cariogenic bacteria, and identifying *S. mutans*. Dental plaque was picked up by pairwise coupling. All processes were conducted by each student, which required their ability to understand the main methods of isolation, culture, and identification of the cariogenic bacteria and the characteristics of the colony of *S. mutans* [11].

1. Sampling and processing of dental plaque: Collecting supragingival plaque, transmitting the sample, using vortex oscillation and ultrasonic oscillation successively to scatter plaque or bacterial clumps, and dispersing plaque specimens by a series of 10 times (the dilution process requires sterile operation).
2. Isolation and cultivation of cariogenic bacteria: Streak inoculating on Mitis-Salivaris-Bacitracin (MSB) agar selective petri dish and culturing oral microbial in anaerobic environment. Selecting typical colonies of *S. mutans* from the original culture plate, then inoculating in Brain Heart infusion (BHI) liquid culture medium under aseptic condition, culturing 24 h in anaerobic environment.
3. Observation of oral microbiology: Observing the original colonies on MSB agar through an anatomical microscope, identifying cariogenic bacteria and non-cariogenic bacteria, recording the colony morphology including morphology, size, thickness, edge, transparency and colour. Gram’s staining bacteria from liquid medium, observing with a microscope. Put 10 μl of the fluid medium into each mannitol, sorbitol, raffinose, melibiose, and arginine sugar tube under aseptic operation for microbial biochemistry identification, then anaerobic incubation for 24 h, and then observing the results.
4. Analysis of the experimental results: According to the score of experimental results to summarize the quantitative data of lab curriculum. According to their reports and open questionnaire to evaluate the qualitative data.

### Lab curriculum grading criteria

Score according to the result of each experiment (Tables 1, 2 and 3) rather than test paper.

**Table 1:** Grading criteria of experiment 1 in evaluating oral microbiology lab curriculum reform for stomatology training at Wuhan University, 2009–2013

Grade	Criterion	Capability evaluation
A	culture cariogenic bacteria successfully	basic ability + professional ability + comprehensive ability + innovation ability are good
B	culture other bacteria	professional ability is inadequate
C	culture no bacteria	basic ability + professional ability are inadequate

**Table 2:** Grading criteria of experiment 2 in evaluating oral microbiology lab curriculum reform for stomatology training at Wuhan University, 2009–2013

Grade	Criterion	Capability evaluation
A	Two copies success	Basic ability is good
B	One copies success	Basic ability is inadequate
C	All failure	Basic ability is poor

**Table 3:** Grading criteria of experiment 3 in evaluation of oral microbiology lab curriculum reform for stomatology training at Wuhan University, 2009–2013

Grade	Criterion	Capability evaluation
A	All success	basic ability + professional ability + comprehensive ability + innovation ability are good
B	Partial success	professional ability is inadequate
C	All failure	basic ability + professional ability are inadequate

Experiment 1: The collection of dental plaque and cariogenic bacteria first separation. This part was the test of basic skills as well as the training of oral clinical professional skills. Acquisition of plaque as the first step was used to train students how to do oral examination. Sterile operation and streak cultivation tested their ability to conduct microbial basic experiments (Table 1).

Experiment 2: Cultivate amplification of *S. mutans*. This part covered the basic operative skills of microbiological experiments. Students who failed to separated *S. mutans* from dental plaque in experiment 1—that is, students who achieved a “C” score—were given new strains to go to the next process (Table 2).

Experiment 3: Biochemical identification of cariogenic bacteria (Table 3).

Finally, we analysed the experimental results and the reasons for success and failure to cultivate the students’ enthusiasm for scientific research. This part reflected the students’ comprehensive ability and innovation ability.

### Students’ evaluation

Part two used qualitative evaluation of students’ reports and open-ended questionnaire, which was designed to assess students’ perceptions of the oral microbiology lab curriculum. We measured students’ perceptions of (1) results of their lab work, (2) reasons for success, (3) lessons of failure, (4) suggestion for this course, and (5) dissatisfaction or discontent about the lab curriculum.

## Results

Both quantitative and qualitative data evaluation of students’ performance and perceptions supported continuing use of the innovative lab teaching. The results supported the study hypotheses.

### Part One

Assessment of student performance in the oral microbiology lab curriculum from 2009 to 2013 are summarized in Table 4.

**Table 4:** Performance data of the oral microbiology lab curriculum (the number of the students and percentage) in evaluating oral microbiology lab curriculum reform for stomatology training at Wuhan University, 2009–2013

	Grade	2009	2010	2011	2012	2013	Total
Experiment 1	A	30(75.0 %)	24(43.6 %)	31(55.4 %)	30(44.8 %)	49(92.5 %)	164(60.5 %)
	B	6(15.0 %)	7(12.7 %)	6(10.7 %)	7(10.4 %)	4(7.5 %)	30(11.1 %)
	C	4(10.0 %)	24(43.6 %)	19(33.9 %)	30(44.8 %)	0(0 %)	77(28.4 %)
Experiment 2	A	39(97.5 %)	33(60.0 %)	47(83.9 %)	66(98.5 %)	40(75.5 %)	225(83.0 %)
	B	1(2.5 %)	16(29.1 %)	9(16.1 %)	1(1.5 %)	7(13.2 %)	34(12.5 %)
	C	0(0 %)	6(10.9 %)	0(0 %)	0(0 %)	6(11.3 %)	12(4.4 %)
Experiment 3	A	37(92.5 %)	34(61.8 %)	47(83.9 %)	59(88.1 %)	44(83.0 %)	221(81.5 %)
	B	3(7.5 %)	18(32.7 %)	5(8.9 %)	0(0 %)	5(9.4 %)	31(11.4 %)
	C	0(0 %)	3(5.5 %)	4(7.1 %)	6(9.0 %)	4(7.5 %)	17(6.3 %)
Total		40	55	56	67	53	271

Experiment 1 was carried out by means of “collection of dental plaque and separation of cariogenic bacteria” to train and assess students’ basic, professional, innovative, and comprehensive abilities. In total, 60.5 % of the 271 students cultivated cariogenic bacteria successfully.

Experiment 2 focused on the microbial pure generation culture. The process was not complicated, focusing on the interaction between students and faculty, and developing the students’ basic experimentation skills and scientific research habits. In total, the 5-year success rate was 83.0 %.

Experiment 3 on “biochemical identification of cariogenic bacteria”, cultivated the students’ basic, professional, innovative, and comprehensive abilities. It helped students to master oral microbiology experiment design and technology. Students reached an 81.2 % 5-year success rate, and the failure rate significantly was 6.3 %.

### Part Two

According to analysis of students’ perceptions of the oral microbiology lab curriculum, the new lab course had many benefits to students and was welcome.

1. Consolidating theory knowledge content

Some students said the following. “When we collected dental plaque for the purpose of cultivating cariogenic bacteria, we recalled the predilection sites of dental caries learned from the theory curriculum and then chose the sites and fissures.” “The colony morphology observation of various types of cariogenic bacteria deepens the image of cariogenic bacteria.” “We associated the four factors theory on dental caries, deepening the understanding of the etiology of dental caries”.

(2) Improving experimental skill

Some relevant students statements follow. “The lab curriculum has strengthened the consciousness of aseptic operation to prevent pollution, such as paying attention to not opening the petri dish cover too much when picking bacteria, inoculation bars need strict sterilisation, operations should be performed on an aseptic operation table, and so on.” “Our streak inoculation techniques have improved. We grasp the bar technique, line intensity, and density well and finally achieve good separation.” “The curriculum has cultivated good experiment habits in us, such as making signature on the petri dish cover in order to prevent the imprinting from influencing observation of the colony.” “The lab operation seems easy for the teacher, but difficult for us. We realise that lab skill comes from practice rather than from high aims but low abilities.”

(3) Cultivating innovative scientific thinking and attitude

Some students who did not cultivate the cariogenic bacteria successfully reflected on it, consulted documents, and communicated with classmates and teachers. The lab curriculum is an efficient way of cultivating the scientific attitude of seeking truth from facts, creativity and curiosity, and cooperative team consciousness. Some students think that the lab curriculum helps to develop interest in scientific research, form a rigorous scientific attitude, become familiar with the process, and pave the way for later study.

(4) Strengthening awareness of oral health

Some students made relevant remarks. “I have no dental caries, but cariogenic bacteria are also cultivated successfully. I recognize the risk of dental caries when my immune defence declined or my oral health is ignored, so, we cannot ever ignore oral hygiene.”; “We practise together and share the process. It contributes to common progress, and we generally like this form of learning”.

## Discussion

Traditional oral microbiology courses consist of lectures only without any practical experience. With the increasing update of oral microbiology, the traditional teaching model was unable to meet the needs of students [4, 12–15]. That is the reason that we introduced a new lab curriculum. The purposes of innovative experimental teaching are: (1) supplementing lectures through clarification and comprehension of theory knowledge and visualisation of abstract concepts; (2) helping students to master experimental skills and relevant safety knowledge of medical microbiology; and (3) helping to cultivate students’ professional skills, scientific research ability, and comprehensive quality and to guide students’ innovative ability and develop their subjective initiative.

Both quantitative and qualitative evaluation support our lab curriculum, which plays an irreplaceable role in training interdisciplinary and innovative talent. In Part One of the study, we retrospectively analysed students’ performance data from the oral microbiology lab curriculum in 2009, 2010, 2011, 2012, and 2013. The success rate in experiment 1 in 2013 was 92.5 %—significantly higher than the other 4 years. This may be because the teaching experience of instructors has been increasing.

Our lab curriculum has four features: −basic skill experiment, professional skill experiment, comprehensive experiment, and innovative experiment. This curriculum actively promotes inquiry and cooperative learning and exploits autonomous and innovative lab teaching content. It is a huge test for students because the experiments go forward one by one while they simultaneously cultivate students’ understanding of the scientific research process. By setting up an experiment, students experience setback, inadequacy, and success which stimulate the desire of higher learning and scientific research and improve basic and professional skills.

Basic skill experiment: This feature refers to the basic operation skills of a microbiology experiment, such as medical laboratory safety protection, preparation of culture medium, medium sterilisation, sterile working technique, operation of the microscope, bacteria dyeing, and bacteria separation. This curriculum requires students to have the experimental skills mentioned above. Basic experiment is the primary requirement of the curriculum, reflecting the most fundamental and essential microbiology experiment operation skills of students.

Professional skill experiment: This lab curriculum highlights professional skills training, such as oral examination, clinical samples of dental plaque, identification of oral bacteria, and so on. It is done to cultivate students’ professional basic skills to adapt to oral medicine developments, while focusing on how to promote the practical application of professional knowledge.

One student said, “In this experiment, I was the lucky one. Although the colony is less on the medium, they are all *S. mutans*. I think it is mainly because of correct sampling. However, we should not do everything with fluky. We should firmly grasp every step of the experiment”.

Comprehensive experiment: This feature pays attention to the training of integrated and innovative abilities. Through the comprehension of basic medical, dental clinical medical, microbiology, caries, periodontal disease, and molecular biology, it widens students’ views and helps them solve problems. For example, if a student does not isolate *S. mutans*, it may be the result of a variety of reasons. Students need to analyse the experiments step by step. “Is the problem of sampling only food debris?” “Is the problem uneven oscillation so that the sample could not be suspended uniformly?” “Is the problem overcrowding inoculation?” And so on. Comprehension as a mix of knowledge from different fields is the advantage of this curriculum.

Innovative experiment: This part emphasises scientific thinking, the cultivation of research ability, and innovation consciousness. Teachers put forward experimental targets. Students practise independently, carrying out special research or comprehensive research based on the above. For example, if they fail to select cariogenic bacteria, students could innovatively groom the non-cariogenic bacteria and compare the difference of the two kinds of bacteria. Innovation is the most interesting aspect of this lab curriculum because it mainly develops students’ thinking, innovation ability, and scientific research ability. For instance, a student reported that “the experiment provides some train of thought for us for after bacteria-related experiments”.

This curriculum requires teachers to have good professional knowledge and research skills. Teachers need to conduct detailed experimental guidance and demonstration, analyse problems, learn lessons, and solve problems. The new lab curriculum needs to have a higher level of scientific research platform. Our course was carried out in a key laboratory for improving the teaching system. This key laboratory is a famous platform in China, named “the State Key Laboratory Breeding Base of Basic Science of Stomatology, Hubei Province & Key Laboratory of Oral Biomedicine (Wuhan University), Ministry of Education”.

## Conclusion

Based on a 5-year quantitative and qualitative appraisal, the oral microbiology lab curriculum seems to reform and improve the quality of oral medicine education and to cultivate high-quality innovative medical talents.
